# The complete chloroplast genome sequence of *Thymus mongolicus* (Labiatae), a special spice plant

**DOI:** 10.1080/23802359.2020.1778573

**Published:** 2020-06-26

**Authors:** Li Huaizhu, Liang Bai, Jiqing Bai, Ping Wang, Caihua Zhou, Dou Lingling, Jiaojiao Jiang, Jun Liu, Qiaoli Wang

**Affiliations:** a School of Chemistry and Chemical Engineering, Xianyang Normal University, Xianyang City, China; b Yulin Agricultural and rural bureau, Yulin City, China; c College of pharmacy, Shaanxi University of Chinese Medicine, Xianyang City, China

**Keywords:** *T. mongolicus*, chloroplast genome, Labiatae

## Abstract

*Thymus mongolicus* is well-known spice plant and resource of traditional Chinese herbal medicine, belonging to the *Thymus* of the Labiatae family. In this study, the whole chloroplast genome of the *T. mongolicus* was sequenced, assembled and annotated, which contains 134 unique genes, including 89 protein-coding genes, 37 tRNA genes and 8 rRNA genes. A maximum likelihood phylogenetic tree based on 21 complete chloroplast genomes revealed that *T. mongolicus* is closely related to *Mentha* genus. The chloroplast genome could be used for species, varieties and medicinal materials identification, genetic engineering and Labiatae germplasm resources protection.

The *Thymus mongolicus* is a well-known spice plant and the resource of traditional Chinese herbal medicine, belonging to the *Thymus* of the Labiatae family. It is widely distributed in North of China and used in traditional Chinese medicine treatment, food health, daily chemical industry, ecological conservation and Mutton cooking (Qiu et al. 2018). The extract of *T. mongholicus* has antioxidative, antibacterial, anti-inflammatory, anticancer and immunity enhanced effects(Chang et al. 2020). However, the biological research of *T. mongholicus* is very few, and there is a little nucleic acid sequences in the Genbank.

To date (5/20/2020), more than 35 different Labiatae species chloroplast genome have been sequenced and deposited into the National Center for Biotechnology Information (NCBI). However, the chloroplast genome of the *T. mongolicus* has not been reported. In this study, we firstly reported the complete chloroplast genomes of *T. mongolicus* based on Illumina Hiseq pair-end sequencing data.

The sample of *T. mongolicus* was collected from Yizheng town, Zhidan county in the Shaanxi Province (Geographic coordinates: 36°38′7.85″N, 110°32′1.28″E; Altitude: 1198 meters), frozen and preserved at Xianyang Normal University. The specimen (No. 610625190628050LY) was deposited in the herbarium of Xianyang Normal University. Total genomic DNA was extracted with modified CTAB method (Stefanova et al. 2013). Genome sequencing was performed by HiSeqX at Biomarker Technologies Corporation. Low quality sequences were filtered with Q30 (base Phred quality score of ≥30). Total high quality reads were mapped to reference (*Mentha longifolia* chloroplast genome: NC_032054.1) using Bowtie2(Langmead and Salzberg 2012) and the mapped reads were extracted and assembled by SPAdes (Bankevich et al. 2012). The assembled chloroplast genome was annotated and manually corrected using Geneious (Kearse et al. 2012), and was deposited into GenBank(accession number NC_046520).

The complete chloroplast genome of *T. mongholicus* is 151,834 bp in length, containing two inverted repeats (IRa and IRb: 25,605 bp), a large single copy (LSC: 82,956 bp), and a small single copy (SSC: 17,668 bp). A total of 134 genes were annotated, including 89 protein-coding genes, 37 tRNA genes, and 8 rRNA genes. The GC content of the complete chloroplast genome is 37.8%.

The Maximum likelihood method were adopted to construct the molecular phylogenetic tree, so as to elucidate the evolutionary relationship of *T. mongolicus.* A total of 21 complete chloroplast genome, including *T. mongolicus* and 19 other Labiatae species were multiple aligned by MAFFT (Kazutaka Katoh et al. 2002). And then, the maximum likelihood phylogenetic tree was generated by RAxML v7.2.8 (Stamatakis 2006) with 1000 bootstrap replicates (Figure 1). The results showed that *T. mongolicus* was closely related to the *Mentha* genus. The evolutionary relationship is similar to the result of morphological classification. Sequencing of the complete chloroplast genome of *T. mongolicus* would lay foundations for *Thymus* genus species and medicinal materials identification, basic biological research, Labiate germplasm resources protection.

**Figure 1.**
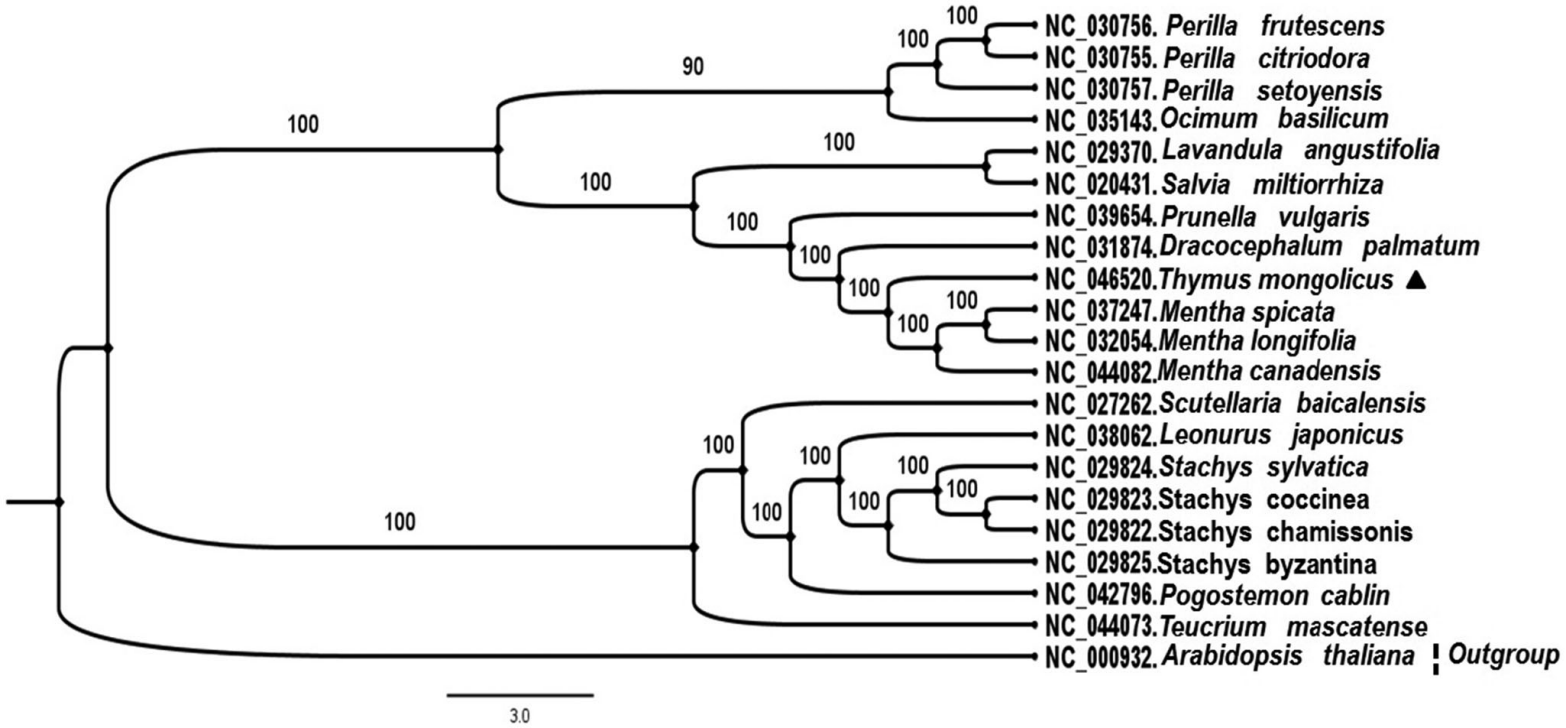
Maximum-likelihood phylogenetic tree base on 21 completely chloroplast genomes. The accession numbers are shown in the figure. Bootstrap support values based on 1000 replicates are displayed on each node. *Arabidopsis thaliana* as outgroup. Marked by a black triangle is *T. mongolicus* in this study.

## Data Availability

The data that support the findings of this study are openly available in GenBank of NCBI at https://www.ncbi.nlm.nih.gov, reference number NC_046520.
